# Functional Assessment of Region-Specific Neglect: Are There Differential Behavioural Consequences of Peripersonal versus Extrapersonal Neglect?

**DOI:** 10.1155/2014/526407

**Published:** 2014-01-29

**Authors:** T. C. W. Nijboer, A. F. ten Brink, M. Kouwenhoven, J. M. A. Visser-Meily

**Affiliations:** ^1^Department of Experimental Psychology, Helmholtz Institute, Utrecht University, Heidelberglaan 2, 3584 CS Utrecht, The Netherlands; ^2^Brain Center Rudolf Magnus and Center of Excellence for Rehabilitation Medicine, University Medical Center Utrecht and De Hoogstraat Rehabilitation, Universiteitsweg 100, 3584 CG Utrecht, The Netherlands; ^3^De Hoogstraat Rehabilitation, Rembrandtkade, Rembrandtkade 10, 3583 TM Utrecht, The Netherlands

## Abstract

*Background.* Region-specific types of neglect (peripersonal and extrapersonal) have been dissociated, yet, differential behavioural consequences are unknown. *Objective.* The aim of the current study was to investigate behavioural consequences at the level of basic activities of daily living of region-specific neglect, using the Catherine Bergego Scale (CBS). *Methods.* 118 stroke patients were screened within the first two weeks after admission to the rehabilitation center for inpatient rehabilitation. *Results.* Patients with peripersonal neglect and patients with neglect for both regions had significantly higher total score on the CBS compared to nonneglect patients. Total scores for patients with extrapersonal neglect were comparable to non-neglect patients. ADL impairments were found across activities (e.g., looking towards one side, forgetting body parts, colliding) for both patients with peripersonal neglect and patients with neglect for both regions. Patients with extrapersonal neglect were only impaired on the item on way finding. *Conclusions.* When diagnosing neglect, it is relevant to distinguish the type of region-specific neglect and, where needed, to adjust the rehabilitation program accordingly. As the CBS is *not* developed to typically measure ADL in extrapersonal neglect, it would be of importance to add other (instrumental) activities that heavily rely on processing information in farther space.

## 1. Introduction

Patients with neglect show impaired or lost awareness for events and items on the contralesional side of space [1, 2]. The clinical manifestations of neglect are heterogeneous and may vary in sensory modality (visual, auditory, and/or tactile), spatial reference frames (egocentric (viewer-based) versus allocentric (stimulus-based) [3, 4]), and regions of space (peripersonal (i.e., within reaching distance, also known as neglect for near space) versus extrapersonal (i.e., beyond reaching distance, also known as neglect for far space [5, 6])). Neglect can be task specific and, therefore, multiple tests that may additionally vary in modality, reference frame, and region of space will increase the likelihood of detecting it.

In relatively recent years, interest has grown in especially region-specificity in neglect, either in single case studies [7–13], small group studies [14–18], or larger group studies [19, 20]. There is now ample evidence that neglect can occur in only one region of space (i.e., peripersonal versus extrapersonal space) or in both regions (i.e., peripersonal and extrapersonal space). In a recent study, it was found in a stroke population admitted in a rehabilitation center that, depending on the test used to assess neglect, 8–22% of patients showed neglect at one distance (i.e., peripersonal or extrapersonal neglect), whereas 11–14% of patients showed neglect for both distances [20]. Additionally, concerning the severity of neglect in general, patients with neglect for both peripersonal and extrapersonal space performed worse on neglect tests, compared to patients with neglect for peripersonal or extrapersonal space only [20].

In general, neglect has been associated with slower and more attenuated recovery patterns of sensory-motor impairment [21] as well as limitations in activities of daily living (ADL) [21–24] compared to nonneglect patients. Neglect has a negative influence on functional independence in self-care, transfers, and locomotion, especially in the subacute phase [24]. Despite ample evidence that peripersonal and extrapersonal neglect can be dissociated, it is unknown whether region-specific neglect has a differential functional outcome, and as such is of relevance to rehabilitation. The aim of the current study is to investigate (behavioural) consequences at the level of basic activities of daily living (ADL) of region-specific neglect, by means of the Catherine Bergego Scale (CBS) [25]. During rehabilitation, it is of great importance to define realistic treatment goals; thus, knowledge of functional consequences of region-specific neglect is critical.

## 2. Methods

### 2.1. Participants

Patients were selected from a stroke patient population consecutively admitted for inpatient rehabilitation to De Hoogstraat Rehabilitation, from March to September 2012. Inclusion criteria were (1) first ever stroke, as revealed by CT or MRI and (2) age between 18 and 85 years. Exclusion criteria were (1) severe deficits in communication and/or understanding and (2) no data on the shape cancellation test available.

### 2.2. Procedure

Neglect screening took place within the first two weeks after admission to the rehabilitation center. The ADL observations were obtained within the same week as the neglect screening. The CBS was filled out by nursing staff in the same week as the neglect screening took place. The study was conducted according to the Code of Conduct for Medical Research of the Council of the Dutch Federation of Medical Scientific Societies. A review procedure by a medical ethics committee was not needed due to the fact that the anonymous data was already routinely collected for usual care.

#### 2.2.1. Measures

*Demographic and Stroke Characteristics.* The patient's medical record was reviewed. The following (relevant) admission-to-rehabilitation data were noted: age, gender, time poststroke, hemisphere of stroke, Mini-Mental State Examination (MMSE), Barthel Index, and Motricity Index.

Cognitive status was screened with the MMSE [26], which tests orientation, memory, attention, calculation, language, and construction functions. Scores vary from 0 (severe cognitive impairments) up to 30 (no cognitive impairments). A score of less than 24 is considered as cognitive impairment.

The Barthel Index [27] measures the extent to which a stroke patient can function independently in their activities of daily living (ADL; i.e., feeding, bathing, grooming, dressing, bowel and bladder control, toileting, chair transfer, ambulation, and stair climbing). Scores range from 0 (completely dependent) up to 20 (completely independent).

The Motricity Index [28] assesses the motor impairment in stroke patients. There are three items for the arms (i.e., pinch grip, elbow flexion, shoulder abduction) as well as three items for the legs (i.e., ankle dorsiflexion, knee extension, hip flexion). Scores range from 0 to 100 (ordinal 6-point scale (i.e., 0, 11, 19, 22, 26, and 33 points) per item +1) for arms and legs separately.

#### 2.2.2. Neuropsychological Neglect Assessment

A neglect screening was administered to all patients. This screening included a shape cancellation test presented at two distances: in the peripersonal condition, stimuli were presented on a monitor at a distance of approximately 30 cm, and in the extrapersonal condition, stimuli were presented at a distance of approximately 120 cm. The order of the tasks and the distance at which a task was first presented were randomized across patients. Stimuli were enlarged in the extrapersonal condition to control for visual angle.

The shape cancellation test consisted of a field of 54 targets shapes (0.6° × 0.6°) among 75 distractor shapes of various sizes (with widths ranging from 0.95° to 2.1° and heights ranging from 0.45° to 0.95°). The stimulus presentation was approximately 18.5° wide and 11° high at both distances. Patients were instructed to find all the target shapes presented on the screen and click on them. A circle appeared around the location of each mouse click and remained on screen during the test. The difference in number of clicked targets between the contralesional and ipsilesional side was used to indicate neglect, that is, an asymmetry of at least two omissions between contralesional and ipsilesional sides [24], and hence categorize patients as neglect or nonneglect.

#### 2.2.3. Behavioural Neglect Assessment

The CBS [25] was used for functional assessment of neglect, as it assesses performance in personal (body parts, body surface), peripersonal, and extrapersonal space, as well as in perceptual, representational, and motor domains, by means of direct observations of self-initiated behaviours in 10 everyday activities, such as grooming, colliding, way finding, and finding belongings. For this study, the original English scale was translated into Dutch [29]. The severity of neglect was rated from 0 to 3 points in each item: 0 = no difficulty, 1 = mild, 2 = moderate, and 3 = severe difficulty, resulting in a range of 0–30.

The internal consistency was excellent (Cronbach's alpha: .92). Excellent correlations between the CBS total score and all item scores were reported (correct item-total correlation range: .63–.90), with the exception of the items on adjusting sleeves/slippers (correct item-total correlation: .40), and paying attention to noise or people addressing the patient from one side (correct item-total correlation: .57), which were adequate.

### 2.3. Statistical Analyses

Spatial specificity was defined in terms of whether a patient showed neglect in a single region of space (i.e., peripersonal or extrapersonal only) or in both regions of space (i.e., peripersonal and extrapersonal) on the shape cancellation test. Four groups were made: no neglect, neglect for peripersonal space, neglect for extrapersonal space, and neglect for both regions of space. Nonparametric tests were used due to the small number of patients per group and noncontinuous variables. Patients with neglect (peripersonal, extrapersonal, and both regions) were compared to patients without neglect on demographic and stroke characteristics as well as CBS total scores using the Mann-Whitney test (2-tailed). Additionally, patients with neglect (peripersonal, extrapersonal, and both regions) were compared to patients without neglect on CBS item scores. In all comparisons, we used Bejamini and Hochberg's [30] method of handling the multiplicity problem in statistical testing controlling the false discovery rate, comparing *p*(*i*) with (*i*/10)∗.05.

## 3. Results

### 3.1. Demographic and Stroke Characteristics

A group of 118 patients (57% male; mean age: 57.1 (SD: 12.2)) were included in the study. Of this group, 37 patients showed neglect. An overview of demographic and stroke characteristics of the nonneglect and neglect patients (as measured with the shape cancellation test) is given in Table 1.

There were no differences between patients with neglect and without neglect, with respect to gender, *U* = 680.5, *Z* = −.01, *P* = .990, age, *U* = 1173.5, *Z* = −1.2, *P* = .225, time poststroke onset, *U* = 1295.5, *Z* = −.45, *P* = .651, aetiology, *U* = 1072.5, *Z* = −.65, *P* = .514, Motricity Index arm, *U* = 927.0, *Z* = −.64, *P* = .523, Motricity Index leg, *U* = 817.0, *Z* = −1.53, *P* = .125, and MMSE, *U* = 612.5, *Z* = −1.64, *P* = .101. Groups differed with respect to hemisphere of stroke, *U* = 853.5, *Z* = −3.27, *P* = .001, with more right-sided brain damage for neglect patients, Barthel Index, *U* = 798.0, *Z* = −2.55, *P* = .011, with lower scores for the neglect patients, indicating more dependence in activities in daily living. Last, groups differed with respect to the total CBS score,*U* = 668.0, *Z* = −4.21, *P* < .001, with higher scores for the neglect patients.

### 3.2. Comparisons between Neglect Groups

#### 3.2.1. Clinical and Stroke Characteristics

With respect to the clinical characteristics (Table 2), there were no differences between patients with peripersonal neglect only and patients without neglect for any of the variables, except for the CBS total score, *U* = 87.0, *Z* = −2.90, *P* = .003. There were no differences between patients with extrapersonal neglect only and patients without neglect for any of the variables. Patients with neglect for both regions of space had significantly lower scores on the Barthel Index compared to patients without neglect, *U* = 328.0, *Z* = −3.29, *P* = .001, and higher CBS total scores, *U* = 373.0, *Z* = −3.75, *P* < .001.

#### 3.2.2. Behavioural Consequences: CBS (Figure 1)

Significant differences between patients with peripersonal neglect and patients without neglect were found for all items of the CBS (*P* < .023), but adjusting clothes,*U* = 93.0, *Z* = −.38, *P* = .707, and cleaning the mouth, *U* = 125.0, *Z* = −1.80, *P* = .072. Significant differences between patients with extrapersonal neglect and patients without neglect were found for way finding only, *U* = 77.0, *Z* = −3.99, *P* < .001. Significant differences between patients with neglect for both regions and patients without neglect were found for all items, but ignoring food on the plate,*U* = 603.0, *Z* = −.99, *P* = .322, forgetting body parts, *U* = 504.0, *Z* = −1.52, *P* = .128, and way finding, *U* = 496.0, *Z* = −1.58, *P* = .114.

## 4. Discussion

The aim of the current study was to investigate behavioural consequences at the level of basic activities of daily living of region-specific neglect, as it is currently unknown whether region-specific neglect has a differential functional outcome, which could be of relevance to rehabilitation.

Within the sample of patients with neglect (31.4% of the original sample), three groups could be dissociated on the basis of performance on a shape cancellation test: patients with peripersonal neglect only, patients with extrapersonal neglect only, and patients with neglect for both regions. Two important differences in behavioural consequences were found between these groups: first, both patients with neglect for peripersonal and patients with neglect for both regions of space were much more impaired in basic activities of daily living compared to patients with extrapersonal neglect only, and second, impairments of patients with extrapersonal neglect appeared to be confined to problems with way finding, whereas the results of patients with peripersonal neglect and neglect for both regions were much more diverse. Interestingly, performances of patients with neglect in both regions did not appear to be a simple add-up of impairments of patients with region-specific neglect. In other words, patients with neglect in both regions were not worse than patients with region-specific neglect (i.e., impairment peripersonal only + impairment extrapersonal only ≠ impairment both regions).

With respect to the demographic and stroke characteristics, it was observed that overall patients with neglect were comparable to patients without neglect with respect to gender, age, time poststroke onset, aetiology, strength of upper and lower extremities, and cognition. They differed, however, with respect to hemisphere of stroke, independence in ADL as measured with the Barthel Index, and neglect behaviour as measured with CBS observations. Additionally, the subgroups were also fairly comparable. Patients with peripersonal neglect as well as patients with neglect in both regions were more impaired as observed with the CBS compared to patients without neglect. Patients with neglect in both regions were also more impaired in basic ADL as measured with the Barthel Index compared to patients without neglect. This suggests that the differences found between groups could not be explained by demographic, motor, or cognitive factors. We cannot, however, rule out the possibility that the right hemisphere is crucial in the observed differences in basic ADL between patients with and without neglect. It is known that neglect is more frequent, severe, and enduring after right hemisphere damage [31]. It might be that impairments in basic ADL are more severe after right hemisphere damage. Additionally, there are differences in both severity as well as functional outcome between left- and right-sided neglect patients, and therefore it would have been interesting to investigate differences in behavioural consequences. In our sample, the sample size of especially the group of right-sided neglect patients was too small to statistically compare outcomes between lesion sites in all subgroups; therefore it remains to be seen whether behavioural neglect is different after left versus right hemisphere lesions.

One of the limitations of this study is the size of the subgroups. Even though the original sample of patients was fair (*n* = 118), categorising patients with neglect (*n* = 37) in region-specific deficits led to small samples (*n* = 8 for neglect confined to either peripersonal or extrapersonal space, *n* = 21 for neglect in both regions of space). Despite this, we feel that our findings are of importance, as no other study has looked into behavioural consequences of region-specific neglect so far. Other studies, with preferable larger samples of patients with region-specific neglect, could further indicate specificity of impairment in ADL. Our study has indicated that further studies into region-specific neglect, with outcome measures at several levels (e.g., function/neuropsychology, activity/ADL, and possibly even participation), are warranted.

Importantly, the results of the current study are based on the outcome of the CBS, which is not developed originally to measure ADL in extrapersonal space. Especially the more instrumental activities of daily living, such as housework, shopping, and transportation (and way finding), rely on processing of and/or interactions with farther space. One may argue that the current outcomes for extrapersonal neglect are maybe underidentified or underanalysed. It would thus be of importance to investigate whether other, maybe more instrumental, activities that heavily rely on processing information in farther space are also impaired in neglect and, as such, whether implications of extrapersonal neglect generalize to other activities. On the other hand, the CBS has some items that could definitely be influenced by the presence of extrapersonal neglect, such as (1) paying attention to noise or people addressing from the left, as both may origin from farther space; (2) colliding with people or objects, as navigation requires integration of information from personal, peripersonal, and extrapersonal space; and (3) way finding, which depends mainly on processing of extrapersonal space. With a general checklist such as the CBS, it is, however, difficult if not impossible to disentangle integration of information from personal, peripersonal, and extrapersonal space. To this end, more fine-grained methods are needed, such as walking trajectories indicating online adjustments in direction and speed when “observing” collisions.

In summary, when diagnosing neglect it is relevant to distinguish the type of region-specific neglect and whenever needed, to adjust the rehabilitation program accordingly. Patients diagnosed with neglect for peripersonal space only and patients with neglect for both regions of space could, for example, receive extra attention or training, since patients in this group need more help in daily activities and experience greater functional difficulties in independently performed activities. Furthermore, focus in rehabilitation in patients with extrapersonal neglect could be more on way finding and other activities whereby spatially distant information is used. This is the first study to indicate neglect behaviour between several types of region-specific neglect, suggesting that distinguishing between these regions is not only interesting for scientific purposes but also for diagnostic and rehabilitation purposes. While no observation scale is yet available to further specify impairment at the level of activities across region-specific neglect, we suggest the use of the CBS as a tool that is sensitive to identification of neglect and its functional consequences next to neuropsychological tests at the level of function.

## Figures and Tables

**Figure 1:**
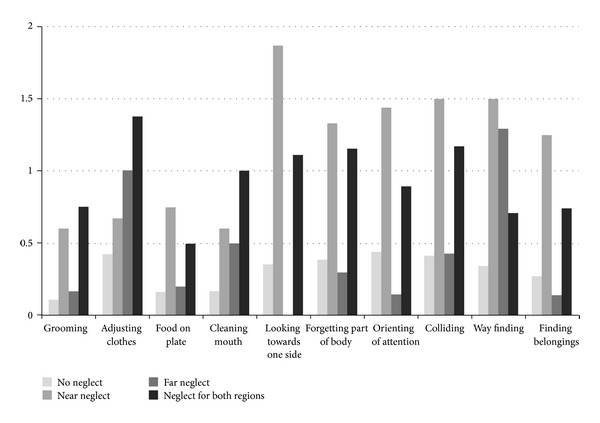
Average scores on all specific items of the CBS, split for groups based on the shape cancellation. Range was from 0 (no neglect) up to 3 (severe neglect).

**Table 1:** Demographical and stroke characteristics per group (nonneglect versus neglect). Groups were made on the basis of performance of a shape cancellation test.

Clinical variables	Nonneglect (SD)	Neglect (SD)
Group size	81	37
Gender (male)	55.3%	55.2%
Age in years	56.0 (12.1)	59.0 (12.7)
Time poststroke in days	35.8 (37.3)	40.8 (32.6)
Hemisphere of stroke (*L*/*R*)	61.1%/34.7%^1^	27.0/67.6%^2^
Aetiology		
Ischemic	79.1%	76.5%
Haemorrhage	11.9%	23.5%
SAH	7.5%	3.7%
Size		
Focal	87.1%	73.5%
Diffuse	12.9%	26.5%
MMSE (0–30)	26.4 (4.1)	24.1 (6.5)
Barthel Index (0–20)	14.6 (4.9)	11.8 (5.5)
Motricity Index arm (0–100)	67.5 (38.2)	60.1 (43.6)
Motricity Index leg (0–100)	74.4 (34.2)	63.6 (37.7)
CBS average total score (0–30)	2.9 (4.9)	9.2 (8.0)

MMSE: Mini-Mental State Examination; CBS: Catherine Bergego Scale; ^1^two patients had bilateral lesions; ^2^one patient had bilateral lesions.

**Table 2:** Clinical characteristics and average total scores on the CBS per group (nonneglect, peripersonal neglect, extrapersonal neglect, both regions, based on a shape cancellation).

Clinical variables	No neglect (SD)	Peripersonal neglect (SD)	Extrapersonal neglect (SD)	Peripersonal and extrapersonal neglect (SD)
Group size	81	8	8	21
Gender (male)	55.3%	62.5%	57.1%	31.8%
Age in years	56.0 (12.1)	63.5 (8.0)	64.6 (13.1).	55.5 (13.3)
Time poststroke in days	35.8 (37.3)			
Hemisphere of stroke (*L*/*R*)	61.1%/34.7%^3^	25%/62.5%^4^	28.6%/71.4%	28.6%/71.4%
Aetiology				
Ischemic	79.1%	62.5%	100%	73.7%
Haemorrhage	11.9%	37.5%	0%	26.3%
SAH	7.5%	0%	0%	
Brain injury				
Focal	87.1%	50%	100%	73.7%
Diffuse	12.9%	50%	0%	26.3%
MMSE (0–30)	26.4 (4.1)	20.6 (10.4)	26.8 (2.9)	24.71 (4.7)
Barthel Index (0–20)	14.6 (4.9)	12.9 (7.0)	15.3 (4.5)	10.0 (4.7)
Motricity Index arm (0–100)	67.5 (38.2)	65.3 (45.5)	78.0 (39.7)	51.7 (44.4)
Motricity Index leg (0–100)	74.4 (34.2)	69.4 (40.5)	80.8 (27.1)	55.1 (38.5)
CBS average total score (0–30)	2.9 (4.9)	12.8 (7.25)	4.2 (4.1)	9.6 (8.6)

MMSE: Mini-Mental State Examination; CBS: Catherine Bergego Scale; ^3^two patients had bilateral lesions; ^4^one patient had bilateral lesions.
